# Synthesis, Characterization, Self-Assembly, and Irritation Studies of Polyglyceryl-10 Caprylates

**DOI:** 10.3390/polym12020294

**Published:** 2020-02-02

**Authors:** Guangyan Zhang, Chenhui Bao, Kaiqiao Fu, Yaolin Lin, Tianlong Li, Huping Yang

**Affiliations:** 1 Hubei Provincial Key Laboratory of Green Materials for Light Industry, Hubei University of Technology, Wuhan 430068, China; xiaochengzikid@163.com (C.B.); li_tianlong2011@163.com (T.L.); 2 Guangzhou KLD New Material Technology Co., Ltd., Guangzhou 510425, China; fukq@gzkeluode.com (K.F.); linyl@gzkeluode.com (Y.L.); yanghp@gzkeluode.com (H.Y.)

**Keywords:** polyglyceryl ester, surfactant, makeup remover, micelles, irritation

## Abstract

1,4-dioxane should be less than or equal to 10 ppm in finished cosmetic products according to the recommendation of the Scientific Committee on Consumer Safety, but it is often generated as a by-product during the manufacturing process of poly(ethylene glycol) (PEG)-based derivatives. In order to avoid the possible risk caused by 1,4-dioxane, it might be a good choice for preparing cosmetic ingredients by using polyglycerin (PG) instead of PEG as a hydrophilic segment. In the present study, polyglyceryl-10 caprylates were synthesized by the esterification reaction between polyglycerin-10 and caprylic acid. FTIR and ^1^H NMR were utilized to confirm the chemical structures of the obtained polyglyceryl-10 caprylates. Light transmittance was availed to investigate the water solubility of polyglyceryl-10 caprylates. The self-assembly behavior, size, and size distribution of polyglyceryl-10 caprylates were investigated by dynamic light scattering. The makeup cleansing effect was also evaluated by in vitro and in vivo methods. Irritation was evaluated by hen’s egg test-chorioallantoic membrane assay (HET-CAM). Results showed that polyglyceryl-10 monocaprylate could self-assemble into nanoparticles in the water at the concentration range of 2.5–10 wt% with a transparent appearance. The diameter of formed nanoparticles was around 100 nm with a narrow particle size distribution around 0.1 at the concentration of 2.5 wt% or 5 wt%. Polyglyceryl-10 monocaprylate exhibited good removal effect against makeup and excellent removal efficacy against pen eyeliner. The irritation of polyglyceryl-10 monocaprylate evaluated by HET-CAM at the concentration of 4 wt% was moderate irritant (irritation score = 8.4), which was lower than that of PEG-6 caprylic/capric glycerides (severe irritant, irritation score = 14.1). Therefore, polyglyceryl-10 monocaprylate might be a promising cosmetic ingredient for transparent makeup removing water.

## 1. Introduction

With the sustained and rapid development of the economy and the improvement of living standards, people are beginning to pay more and more attention to their appearances. The sales volume of the makeup market keeps growing sustainably in the past decades. However, if the makeup cannot be cleaned from the skin in depth, it will accumulate on the skin surface to affect skin metabolism and cause skin problems, such as clogged pores and contact dermatitis [1,2,3]. Therefore, makeup removing process has become an indispensable part of daily life for people who apply makeup products every day, and the research and development of safe makeup remover products are important [4,5,6].

Now, makeup remover products mainly include cleansing oil, cleansing gel, and makeup removing water. Among them, the makeup removing water is popular in recent years due to its transparent appearance, convenient use, being refreshing, and non-sticky. In fact, makeup removing water consists of many nano-scaled micelles, although their appearances are transparent. At present, the cosmetic ingredients that can be used for transparent makeup removing water mainly include polyethylene glycol (PEG)-6 caprylic/capric glycerides (P6CCG), poloxamer 184 (poly(propylene glycol)-poly(ethylene glycol) block copolymer), and olive oil PEG esters. These ingredients can form nano-scaled micelles in water with a transparent appearance at a typical additive amount of 2–10 wt%. However, studies have found that it may produce 1,4-dioxane as a by-product during the manufacturing process of PEG-based derivatives [7]. 1,4-dioxane, which was first listed in the Second Annual Report on Carcinogens (1981), had been listed as “reasonably anticipated to be human carcinogens” again in 14th Report on Carcinogens released by U.S. Department of Health and Human Services (National Toxicology Program) in 2016 [8,9]. Meanwhile, the Scientific Committee on Consumer Safety (SCCS) has suggested that the acceptable trace level of 1,4-dioxane is less than or equal to 10 ppm in finished cosmetic products [10]. Therefore, developing novel cosmetic ingredients without the PEG segment for transparent makeup removing water shows huge potential and a great prospect.

Polyglycerin has received many concerns because of its flexible polyether backbone and biocompatibility and can be synthesized using glycerol as raw material by condensation polymerization using acid or base as catalyzer [11,12]. Polyglycerin has numerous hydrophilic hydroxyl groups, which can be modified to prepare different polyglyceryl derivatives for various applications, such as surfactants [13,14,15], food additives [16,17,18], pharmaceuticals [19,20,21], and cosmetics [22,23]. Polyglyceryl fatty acid esters are one of the most investigated polyglyceryl derivatives [24,25], and their chemical and physical properties can be regulated by adjusting the degree of esterification [26]. Moreover, the safety of polyglyceryl esters was also evaluated in a 2-years dietary study, and results showed that they were not carcinogenic and did not produce any adverse effects (test concentration: 5%) [27]. In addition, both glycerol and fatty acids are bio-based raw materials, which can be obtained from plant oils or fats via hydrolysis reaction. Thus, polyglyceryl fatty acid esters are also important biopolymers, which meet the requirements of safety, bio-based economy, and sustainable development.

PEG-6 caprylic/capric glycerides, which is one kind of nonionic surfactant based on poly(ethylene glycol), can self-assemble into nano-micelles in water with the transparent appearance and has been widely applied in makeup removing water. However, previous work showed that polyglyceryl monolaurates exhibited better surfactant properties than their counterpart poly(ethylene glycol) surfactants due to the presence of a secondary hydroxyl group in each glycerol unit [28]. In addition, polyglyceryl esters have another important advantage that they are free of 1,4-dioxane comparing with PEG-based derivatives. Therefore, polyglyceryl fatty acid esters based on caprylic acid (C8) and/or capric acid (C10) may be a better cosmetic ingredient for makeup removing water than PEG-6 caprylic/capric glycerides if the appearances of their aqueous solutions are transparent.

In fact, there are some polyglyceryl fatty acid esters based on caprylic acid and/or capric acid, such as polyglyceryl-10 caprylate/caprate (e.g., Polyaldo^®^ 10-1-CC, Lonza, Basel, Switzerland), polyglyceryl-6 caprylate (e.g., TEGOSOFT PC 41 MB, Evonik, Essen, Germany), and polyglyceryl-4 caprate (e.g., TEGO Solve 90 MB, Evonik), which have been already used in cosmetics. However, they are usually applied as a solubilizer to solubilize fragrance or as a high-performing foam boost to improve foaming in cosmetics. Additionally, for preparing makeup cleansing or removing cosmetics with a clear or translucent appearance, polyglyceryl-4 caprate needs to compound with other ingredients (e.g., fatty acid esters of sucrose), and the ratio of sucrose fatty acid esters to polyglyceryl fatty acid esters should be greater than or equal to 0.06 [29].

As is well-known, the chemical and physical properties of polyglyceryl fatty acid esters are related to the degree of esterification, the carbon numbers of fatty acids, and the degree of polymerization (DP) of polyglycerin. Thus, the properties of polyglyceryl-10 caprylates may be different from that of polyglyceryl-10 caprylate/caprate (e.g., Polyaldo^®^ 10-1-CC) or other polyglyceryl caprylates/caprates with different DP of polyglycerin. So far, no work has reported the relationship between the appearances of polyglyceryl-10 caprylates aqueous solutions and the degree of esterification. The removal efficacies against makeup cosmetics and irritations of transparent polyglyceryl-10 caprylates aqueous solutions have also been unrevealed. The purpose of the present study was to prepare polyglyceryl-10 caprylates with varying esterification degrees and investigate the appearances of their aqueous solutions, self-assembly behaviors in water, and irritations. The in vitro and in vivo cleansing efficacies were also evaluated.

## 2. Materials and Methods

### 2.1. Materials

Polyglycerin-10 (PG-10) was purchased from Yueyang Keluode United Chemical Industry Co., Ltd. (Yueyang, China) (hydroxyl value: 891 mg KOH/g; electrical conductivity of 10 wt% PG-10 aqueous solution: 58 μs/cm). Caprylic acid was purchased from Adamas Reagent Co., Ltd. (Shanghai, China) and used as received. HND-27 solid super-acid catalyst was purchased from Jiangyin Nanda Synthetic Co., Ltd. (Jiangyin, China). Poloxamer 184 was manufactured by BASF China (Shanghai, China). PEG-6 caprylic/capric glycerides and olive oil PEG-8 esters were provided by Yueyang Keluode United Chemical Industry Co., Ltd. (Yueyang, China). All other reagents were of analytical grade.

### 2.2. Synthesis of Polyglyceryl-10 Caprylates

Polyglyceryl-10 caprylates, denoted as PGCE, were synthesized by the esterification reaction between polyglycerin-10 and varying amounts of caprylic acid. Polyglycerin-10 (75.8 g, 0.1 mol) was placed in a 150 mL flask, and then varying amounts of caprylic acid were added. Solid super-acid HND-27 (2.28 g) was added to catalyze the esterification reaction. The mixture was heated and remained at 150 °C with continuous stirring under a vacuum of −0.095 Mpa until the appearance of the reaction mixture changed from heterogeneous turbidity to homogeneous transparency. Then, the mixture was kept for another 1 h reaction. Next, the reaction mixture was heated to 180 °C for 30 min to remove unreacted caprylic acid. At last, the reaction mixture was cooled to room temperature and then filtered with a vacuum pump to remove catalyzer, and light yellow viscous liquid polyglyceryl-10 caprylates were obtained.

### 2.3. Structural Characterizations

Fourier transformed infrared (FTIR) spectra were determined with Nicolet 6700 (Thermo Fisher, Hampton, NH, USA). Proton nuclear magnetic resonance (^1^H NMR) spectra were recorded with Bruker AVANCE III HD spectrometer (400 MHz) (Bruker, Billerica, MA, USA) in DMSO-d_6_.

### 2.4. Solubility in Water

PGCE (1 g) was weighed and then diluted with deionized (DI) water to the concentration of 10 wt%, 7.5 wt%, 5 wt%, 2.5 wt%, and 1.25%, respectively. The appearances of obtained PGCE aqueous solutions were recorded with a camera in glass bottles with a diameter of 18 mm and a height of 65 mm. The light transmittances of PGCE aqueous solutions were measured with Shimadzu UV-2550 UV/Vis spectrometer (Shimadzu, Kyoto, Japan) at 500 nm. The light transmittance of DI water was defined as 100%.

### 2.5. Self-Assembly Behavior

The self-assembly behaviors of polyglyceryl-10 caprylates in DI water were determined by dynamic light scattering (Nano ZS ZEN3600, Malvern Instruments, Westborough, MA, USA) with a fixed scattering angle of 173° at 25 °C.

### 2.6. Cleansing Efficacy

Pen eyeliner (Super black fixed liner, Mistine, Bangkok, Thailand; Batch No: 9KA; EXP: 20221101) was used to evaluate the in vitro makeup removal efficacy by the published protocol [30,31] with slight modification. Briefly, pen eyeliner was weighted accurately and then diluted with varying amounts of acetonitrile in a serial concentration of 0.5–2.5 mg/mL. The mixtures of pen eyeliner and acetonitrile were sonicated (5 min) and then filtered with a 220 nm filter. The obtained filtrates were determined by Shimadzu UV-2550 UV/Vis spectrometer (Shimadzu, Kyoto, Japan) at the wavelength range of 200–400 nm to record their UV absorption spectra. The validation was examined at the obtained specific wavelengths by linear regression.

In vitro cleansing efficacy of PGCE was evaluated by the following procedure. Pen eyeliner (0.045 g) was accurately weighed onto a clean glass (25 mm × 75 mm). Five minutes later, PGCE aqueous solution (0.090 g) with different concentrations of 2.5–10 wt% was added and then wiped by the cotton sheet. Next, the makeup on the wiped cotton sheet was extracted with 30 mL acetonitrile by sonicating extraction, and the mixture was filtered for the following UV spectroanalysis. At last, the absorbance was recorded to calculate the makeup cleansing efficacy. The blank sample without PGCE was measured as a control. P6CCG was also evaluated, as mentioned above, for comparison.

Oily marker pen (White geese, Cixi, China), lip fluid (Mini point super shine lip fluid, Miniso, Guangzhou, China), and pen eyeliner (Double-head eyeliner, Miniso, China) were used to evaluate in vivo makeup removal efficacy. Ten Chinese female subjects (20–40 years old) who applied makeup products for every day were involved in this study. All recruited volunteers were informed about the study. A total of 24 samples (two ingredients: PGCE-1 and P6CCG; four aqueous solutions with different concentrations for each ingredient; triplicate samples for each concentration) labeled with different codes were provided to the above ten subjects for evaluating the makeup removal effects of PGCE-1 and P6CCG. Makeup removal score was graded from 0 to 10 by cleanliness (10, corresponding to no residual makeup in visual).

### 2.7. Irritation

Irritations of polyglyceryl-10 caprylates and comparison samples (PEG-6 caprylic/capric glycerides, poloxamer 184, and olive oil PEG-8 esters) were evaluated by hen’s egg test-chorioallantoic membrane (HET-CAM) in vitro assay [32]. A 0.9 wt% NaCl solution was used as negative control; 0.1 mol/L NaOH solution (4 wt%) was used as a positive control. Irritation score (IS) was calculated using the following formula [33]:

(1) $$IS=5\times \frac{\left(301-\sec H\right)}{300}+7\times \frac{\left(301-\mathrm{secL}\right)}{300}+9\times \frac{\left(301-\sec C\right)}{300}$$

where secH (hemorrhage time) = observed start time of hemorrhage on chorioallantoic membrane (seconds); secL (vessel lysis time) = observed start of vessel lysis on chorioallantoic membrane (seconds); secC (coagulation time) = observed start of coagulation on chorioallantoic membrane (seconds). Irritation category according to IS range evaluated by HET-CAM is given in Table 1.

## 3. Results

PEG-6 caprylic/capric glycerides is a mixture of polyethylene glycol derivative, which contains mono-, di-, and triglycerides of caprylic/capric acids with an average of six ethylene oxide structural units in one PEG-6 caprylic/capric glycerides molecule [34]. The hydrophile-lipophile balance (HLB) value of PEG-6 caprylic monoglyceride calculated by Griffin’s method is around 14 [35]. In previous work, results showed that polyglyceryl-5 (number-average degree of polymerization of five) monoesters and diesters could form an oil-in-water (O/W) emulsion, polyglyceryl-5 triesters and tetraesters could form both O/W and water-in-oil (W/O) emulsion depending on the carbon numbers of fatty acid (6-18 carbons), and polyglyceryl-5 heptaesters could form W/O emulsion [26]. However, the irritations of the obtained polyglyceryl-5 monocaprylate and dicaprylate and the appearances of their aqueous solutions were not investigated, although the reported HLB values of polyglyceryl-5 monocaprylate (15.4) and dicaprylate (12.2) were close to that of PEG-6 caprylic monoglyceride.

**Scheme 1:**
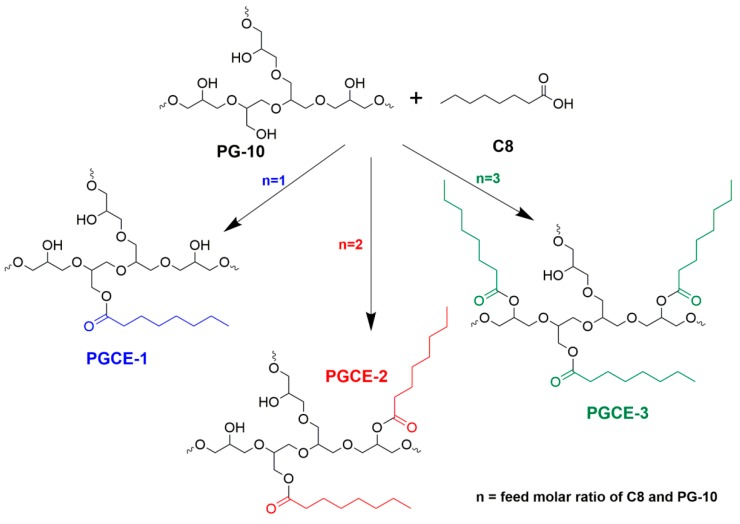
Synthesis routes toward polyglyceryl-10 caprylates.

According to Griffin’s method, the HLB value is proportional to the molecular mass ratio of the hydrophilic portion and the whole molecule. Therefore, the difference of HLB value between polyglyceryl monoester and polyglyceryl diester would decrease with increasing the molecular weight of hydrophilic polyglycerin when the same fatty acid was used as a hydrophobic portion to prepare polyglyceryl fatty acid esters. In order to adjust the HLB value of polyglyceryl caprylates more finely, polyglycerin-10 that has a higher degree of polymerization than polyglycerin-5 was chosen to synthesize polyglyceryl-10 monocaprylate (PGCE-1), dicaprylate (PGCE-2), and tricaprylate (PGCE-3), as shown in Scheme 1, to investigate their properties, including the appearances of their aqueous solutions, self-assembly behaviors in water, in vitro cleansing efficacy, in vivo makeup removal efficacy, and irritations.

### 3.1. Structural Characterization of Polyglyceryl-10 Caprylates

**Figure 1:**
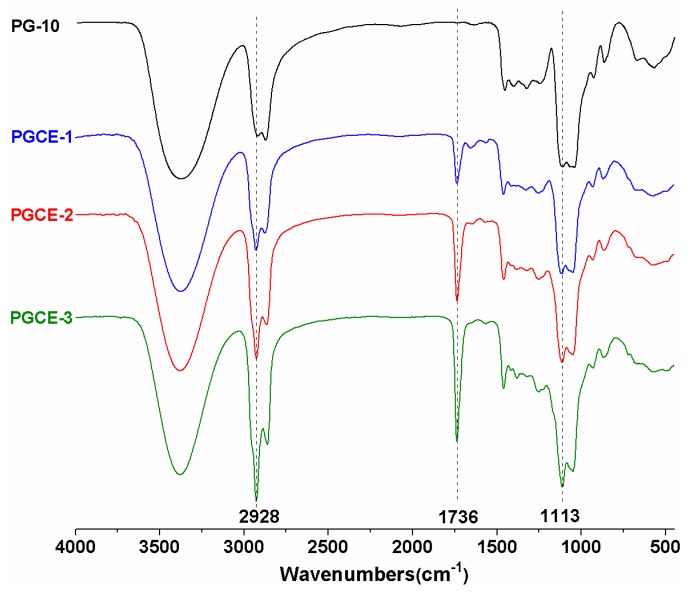
Fourier transformed infrared (FTIR) spectra of polyglycerin-10 (PG-10) and polyglyceryl-10 caprylates (PGCE) in KBr.

FTIR spectra of PG-10 and polyglyceryl-10 caprylates are shown in Figure 1. The strong wide absorption band at 3000–3600 cm^−1^ was ascribed to the stretching vibrations of –OH, and the absorption band at 1000–1200 cm^−1^ was mainly ascribed to the stretching vibrations of –C–O–C– in PG-10 and polyglyceryl-10 caprylates. As could be seen from Figure 1, a new absorption peak at 1736 cm^−1^ was observed in the FTIR spectra of polyglyceryl-10 caprylates comparing to that of PG-10, which implied the formation of the ester bond between polyglycerin-10 and caprylic acid. Additionally, the absorption intensities of peaks at 2928 cm^−1^ (corresponding to –CH_2_– groups), 1736 cm^−1^ (corresponding to >C=O in ester linkages), and 1113 cm^−1^ (corresponding to –C-O-C- in ester linkages) in polyglyceryl-10 caprylates were strengthened with increasing the feed molar ratio of caprylic acid/PG-10. This indicated that the contents of –CH_2_– groups and ester linkages increased after the esterification reaction. This might be ascribed to the successful introduction of caprylic acid (including six methylene groups in one caprylic moiety) to PG-10. Meanwhile, no obvious absorption peak at 1700 cm^−1^ (corresponding to –COOH of caprylic acid) was observed, meaning the residue of unreacted caprylic acid was negligible.

**Figure 2:**
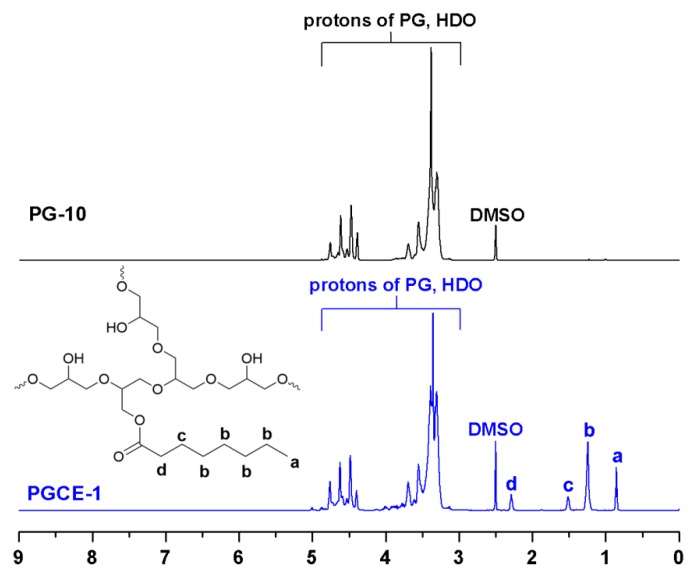
The proton nuclear magnetic resonance (^1^H NMR) spectra of PG-10 and PGCE-1 in dimethyl sulfoxide-d_6_.

^1^H NMR spectra of PG-10 and PGCE-1 are shown in Figure 2. Compared with the ^1^H NMR spectrum of PG-10, four new proton peaks—a (0.85, t, 3H (CH_3_)), b (1.24, m, 8H (CH_2_)), c (1.51, m, 2H (β-CH_2_)), and d (2.25, m, 2H (α-CH_2_))—were observed from the ^1^H NMR spectrum of PGCE-1. Moreover, no obvious proton signal was detected above 9 ppm in the ^1^H NMR spectrum of PGCE-1, which also implied that no obvious unreacted caprylic acid was detected.

The chemical features of polyglyceryl-10 caprylates are summarized in Table 2. As shown in Table 2, the acid values of polyglyceryl-10 caprylates were very low, which further confirmed that the residue of unreacted caprylic acid was negligible. The hydroxyl values of the obtained polyglyceryl-10 caprylates decreased with increasing the feed molar ratio of caprylic acid/PG-10. On the contrary, the saponification values of the obtained polyglyceryl-10 caprylates increased with increasing the feed molar ratio of caprylic acid/PG-10. With the analysis results of FTIR, ^1^H NMR, and chemical features taken together, it could be concluded that the esterification reaction between polyglycerin-10 and caprylic acid was successful.

### 3.2. The Appearance of Polyglyceryl-10 Caprylates in Water

**Figure 3:**
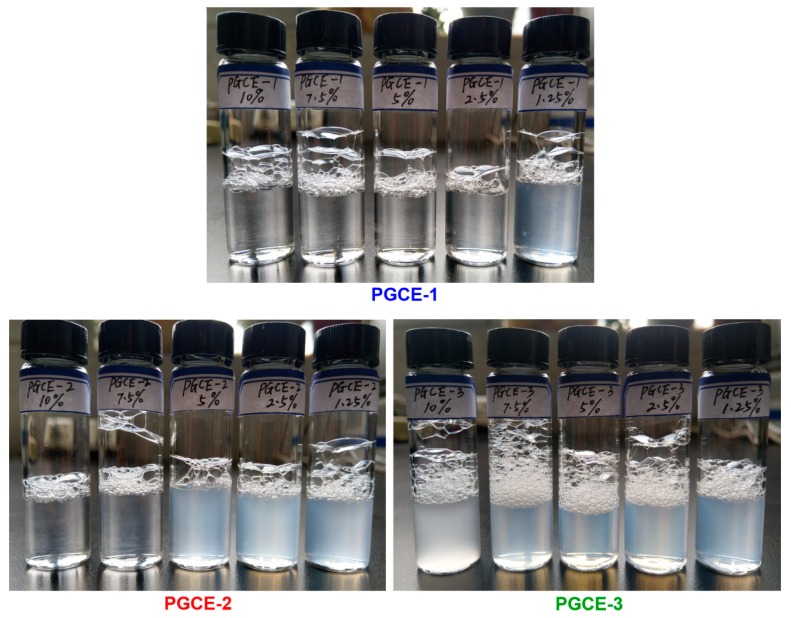
The appearance of polyglyceryl-10 caprylates in the water at different concentrations.

The appearances of polyglyceryl-10 caprylates in water were investigated at the concentration range of 1.25–10 wt%, as shown in Figure 3. The appearances of PGCE-1 (monoester) aqueous solutions were transparent at the concentration range of 2.5–10 wt%, but the appearance became semi-transparent at the concentration of 1.25 wt%. The appearances of PGCE-2 (diester) aqueous solutions were transparent when the concentration of PGCE-2 was more than or equal to 7.5 wt%, while they became semi-transparent when the concentration was equal to or lower than 5 wt%. However, the appearances of PGCE-3 (triester) aqueous solutions were turbid at all tested concentrations.

The aqueous solutions of polyglyceryl-10 caprylates were also investigated by transmittance and summarized in Table 3. DI water was used as a control, and its transmittance was defined as 100%. Transmittance results of polyglyceryl-10 caprylates consisted of their appearances. Interestingly, the transmittances of PGCE-2 aqueous solutions decreased with the decrease of PGCE-2 concentration at the range of 2.5–10 wt%, but the transmittance at the concentration of 1.25 wt% (56.9%) was higher than that at the concentration of 2.5 wt% (43.2%). This might be ascribed to the low content of PGCE-2 in the water at the concentration of 1.25 wt%. The interaction between PGCE-3 and water was weak because of its high hydrophobic property, which might be the main reason for the non-uniform dispersion of PGCE-3 in water. In addition, PGCE-3 aqueous solution formed two separated layers after standing for 2 days at the concentration of 10 wt%. Thus, the change of transmittances was disordered for PGCE-3 at the concentration range of 1.25–10 wt%.

Based on the above appearances and transmittance results of polyglyceryl-10 caprylates aqueous solutions, PGCE-1 might be suitable for the application in makeup removing water at the typical additive amount range of 2.5–10 wt%. Thus, PGCE-1 was selected for further self-assembly behavior and irritation investigations.

### 3.3. Self-Assembly Behavior

**Figure 4:**
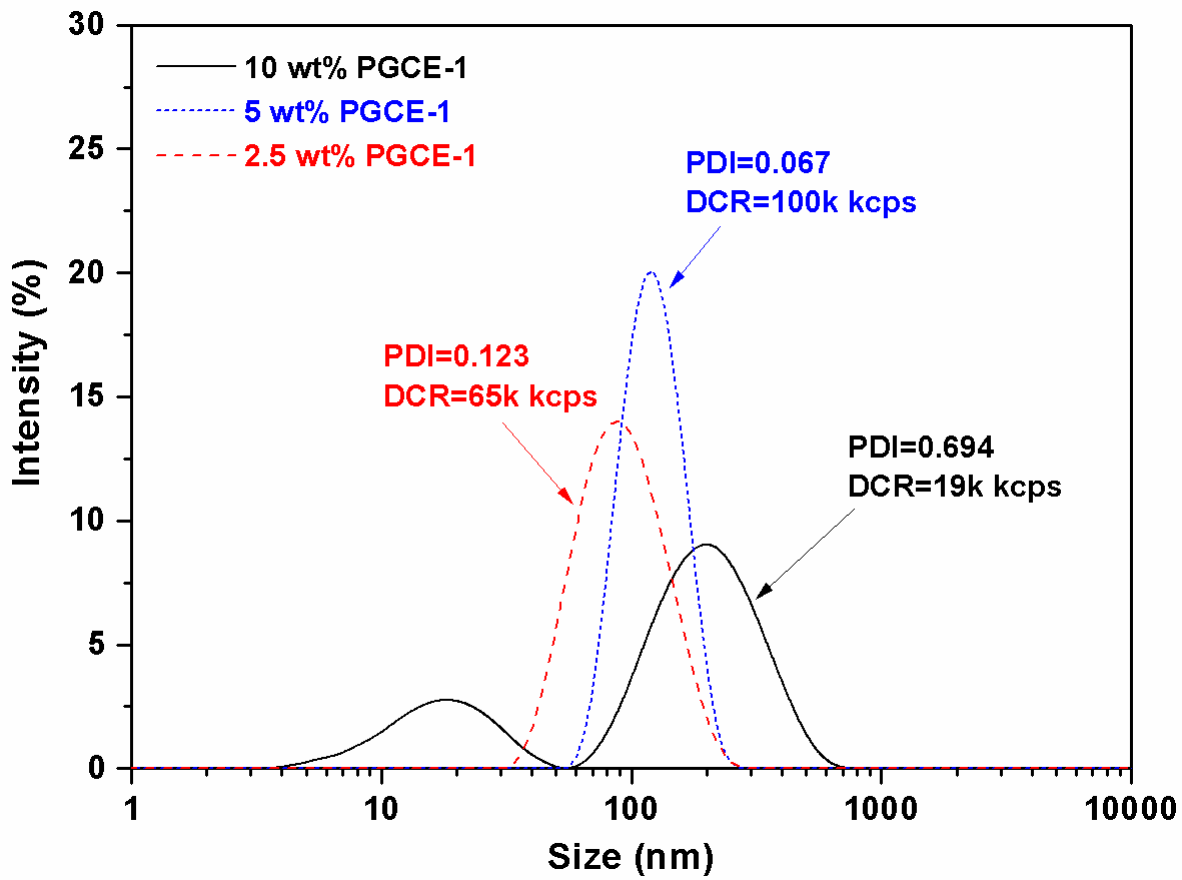
Diameters, polydispersity index (PDI) of size distributions, and scattering intensities (derived count rate: DCR) of PGCE-1 aqueous solutions with different concentrations.

PGCE-1 aqueous solutions were further investigated by dynamic light scattering (DLS). DLS results (Figure 4) showed that the size and scattering intensity of PGCE-1 aqueous solution at the concentration of 2.5 wt% was around 80 nm with a narrow size distribution (polydispersity index (PDI) = 0.123) and 65,000 kcps, respectively. When the concentration of PGCE-1 was raised to 5 wt%, the size and scattering intensity increased to about 110 nm (PDI = 0.067) and 100,000 kcps, respectively. This indicated that PGCE-1 could self-assemble into uniform nano-scaled micelles in water at both concentrations of 2.5 wt% and 5 wt%.

However, when the concentration of PGCE-1 was raised to 10 wt%, the particle size distribution became very wide (PDI = 0.694), and the scattering intensity decreased to 19,000 kcps. As shown in Figure 4, some PGCE-1 molecules self-assembled into small particles with a diameter of 20 nm in water; other PGCE-1 molecules aggregated into big particles with a diameter of around 200 nm. Thus, the size distribution of PGCE-1 at the concentration of 10 wt% obviously became wider than that at the concentration of 2.5 wt% or 5 wt%.

Additionally, the decrease of scattering intensity might also be related to the aggregation behavior of PGCE-1 molecules. Because a majority of PGCE-1 molecules aggregated to form big particles in aqueous solution, the particle concentration that had a positive correlation with scattering intensity might decrease at the concentration of 10 wt%, thus causing low scattering intensity.

### 3.4. Makeup Cleansing Efficacy

**Figure 5:**
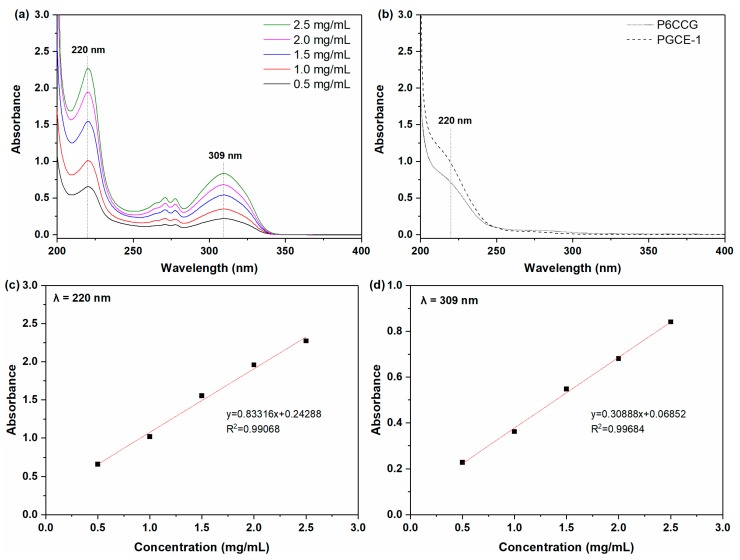
UV spectra of (**a**) pen eyeliner and (**b**) PGCE-1 (1 wt%) and polyethylene glycol (PEG)-6 caprylic/capric glycerides (P6CCG) (1 wt%) in acetonitrile; standard curves for removal efficacy evaluation against pen eyeliner at (**c**) 220 nm and (**d**) 309 nm.

UV spectra of pen eyeliner in acetonitrile at different concentrations are shown in Figure 5a. It could be seen from Figure 5a that there were two absorption peaks at 220 nm and 309 nm, respectively. Although the linearities of pen eyeliner analysis at both 220 nm (Figure 5c) and 309 nm (Figure 5d) were greater than 0.990, the absorption peak at 309 nm was chosen for the next makeup cleansing efficacy test to avoid the UV absorption interference of PGCE-1 or P6CCG to pen eyeliner at 220 nm (Figure 5b shows that both PGCE-1 and P6CCG had UV absorption at 220 nm).

Cleansing efficacies of PGCE-1 aqueous solution against pen eyeliner were evaluated by UV at 309 nm and summarized in Table 4. Results showed that PGCE-1 exhibited better cleansing efficacy than P6CCG at all tested concentrations. This might be ascribed to the greater interaction between PGCE-1 and polymeric ingredients in pen eyeliner, such as acrylates/ethylhexyl acrylate cross-polymer and ammonium acrylates copolymer. The higher content of the hydroxyl group in PGCE-1 than P6CCG might induce more formation of hydrogen bonds between PGCE-1 and acrylates copolymers, which enhanced the interaction between PGCE-1 and pen eyeliner.

**Figure 6:**
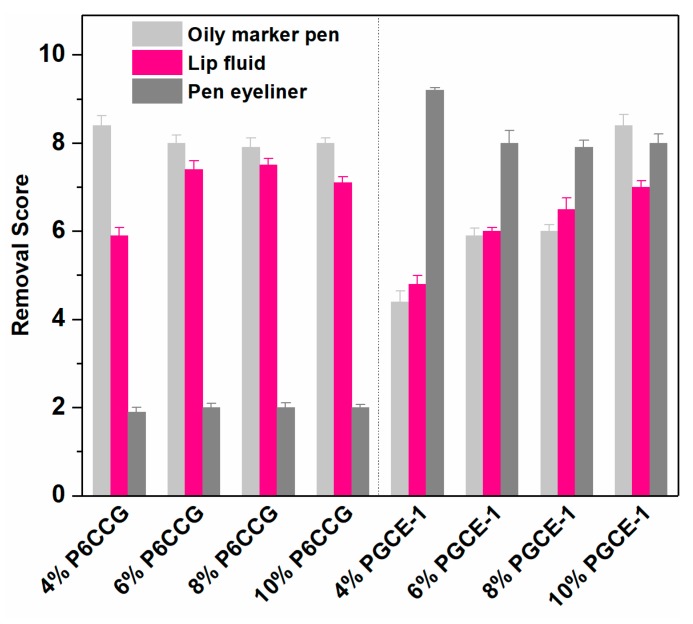
In vivo removal efficacies of P6CCG and PGCE-1 aqueous solution.

In vivo makeup removal results (Figure 6) showed that both P6CCG and PGCE-1 had good makeup removal effects, but P6CCG had better makeup removal efficacies than PGCE-1 against oily marker pen and lip fluid at the concentration range of 4%–8%. However, PGCE-1 exhibited much better makeup removal effects against pen eyeliner than P6CCG at all evaluated concentrations, which was consistent with the in vitro cleansing efficacy results against pen eyeliner. Interestingly, in all evaluated PGCE-1 aqueous solutions, the sample that had the lowest concentration showed best makeup cleansing effects in both in vitro and in vivo makeup removal tests. This might be ascribed to the smaller particle size at lower PGCE-1 concentration (Figure 4), which could promote the permeation of PGCE-1 micelles into polymeric ingredients of pen eyeliner.

### 3.5. Irritation

Irritation of PGCE-1 was evaluated by HET-CAM. P6CCG, olive oil PEG-8 esters, and poloxamer 184 were also evaluated for comparison. A 0.9 wt% NaCl solution and 0.1 mol/L NaOH solution (4 wt%) were used as negative control and positive control to identify the reliability of the experiments, respectively.

After contacting with chicken embryo chorioallantoic membrane for 60 s, 0.1 mol/L NaOH solution exhibited severe irritation because many large blood clots were observed (Figure S1f); while vessel lysis, hemorrhage, and coagulation were not observed for 0.9 wt% NaCl solution (Figure S1e). For 4 wt% PGCE-1 solution, only small spotty hemorrhages were observed (Figure S1a); for 4 wt% P6CCG solution, obvious vessel lysis, large scale hemorrhages, and small blood clots were observed (Figure S1b); for both olive oil PEG-8 esters (Figure S1c) and Poloxamer 184 (Figure S1d), no obvious vessel lysis and blood clots were observed. Thus, it could be seen from Figure S1 that the irritation of PGCE-1 might be comparable to that of olive oil PEG-8 esters and Poloxamer 184, but much lower than that of P6CCG.

The irritation scores of 0.9 wt% NaCl solution and 0.1 mol/L NaOH solutions were 0 and 18.5, respectively, which satisfied the criteria for an acceptable test. More samples were evaluated by HET-CAM, and their irritation scores are summarized in Table 5. As shown in Table 5, the irritation score of PGCE-1 at the concentration of 4 wt% was 8.4 (moderate irritant), which was much lower than that of PEG-6 caprylic/capric glycerides (irritation score: 14.1, severe irritant) and similar to that of olive oil PEG-8 esters (irritation score: 8.2, moderate irritant). At the concentration of 2 wt%, the irritation of PGCE-1 was also much lower than that of P6CCG (Table 5). The order of irritation of four tested samples was P6CCG > PGCE-1, olive oil PEG-8 esters > Poloxamer 184.

## 4. Conclusions

In this work, polyglycerin-10 was chosen to react with caprylic acid for preparing polyglyceryl-10 caprylates, including monoester, diester, and triester. The chemical structures of the obtained polyglyceryl-10 caprylates were characterized and confirmed by FTIR and ^1^H NMR. The appearances and light transmittances of polyglyceryl-10 caprylates in water were investigated at the concentration range of 1.25–10 wt%, and the results showed that the aqueous solutions of polyglyceryl-10 monocaprylate had transparent appearances at the concentration range of 2.5–10 wt%. Dynamic light scattering results showed that polyglyceryl-10 monocaprylate could self-assemble into nano-scaled micelles with a diameter of around 100 nm and narrow particle size distribution (PDI around 0.1) at the concentration of 2.5 wt% and 5 wt%. In vitro and in vivo makeup cleansing results showed that polyglyceryl-10 monocaprylate had a good removal effect against makeup and exhibited much better removal efficacy than P6CCG against pen eyeliner. Irritation results evaluated by HET-CAM showed that the irritation of polyglyceryl-10 monocaprylate (moderate irritant) was comparable with olive oil PEG-8 esters and much lower than that of PEG-6 caprylic/capric glycerides (severe irritant). Therefore, polyglyceryl-10 monocaprylate might be a promising safe cosmetic ingredient for transparent makeup removing water.

## 5. Patents

This research supported Chinese patent CN201810725737.8 (Title: Transparent Water-based Makeup Remover Raw Material and Preparation Method Thereof).

## Figures and Tables

**Table 1:** Irritation category according to the irritation score range evaluated by hen’s egg test-chorioallantoic membrane assay (HET-CAM).

IS* Range	Irritation Category
IS < 1	Non-irritant
1 ≤ IS < 5	Slight irritant
5 ≤ IS ≤ 9	Moderate irritant
IS ≥ 9	Severe irritant

* IS = Irritation score

**Table 2:** Chemical features of polyglyceryl-10 caprylates.

Sample	Feed Molar Ratio (C8: PG-10*)	Hydroxyl Value, mg KOH/g	Acid Value, mg KOH/g	Saponification Value, mg KOH/g
PGCE*-1	1:1	711	0.8	58
PGCE-2	2:1	574	1.4	105
PGCE-3	3:1	489	1.3	132

* PG-10 = Polyglycerin-10; PGCE = Polyglyceryl-10 caprylates.

**Table 3:** Transmittances of polyglyceryl-10 caprylates aqueous solutions at different concentrations.

Samples	Concentrations (wt%)				
	10	7.5	5	2.5	1.25
PGCE-1	98.9%	98.1%	96.3%	95.4%	76.7%
PGCE-2	96.8%	95.8%	63.1%	43.2%	56.9%
PGCE-3	3.7%	41.7%	49.2%	36.1%	48.8%

**Table 4:** In vitro cleansing efficacy of PGCE-1 and polyethylene glycol (PEG)-6 caprylic/capric glycerides (P6CCG) against pen eyeliner.

Remover	Concentration (wt%)	Absorbance	Amount (mg/mL)		Cleansing Efficacy (%)
			Added	Found	
Blank	0	0.22 ± 0.01	1.5 ± 0	0.51 ± 0.01	34.51 ± 0.01
PGCE-1	2.5	0.38 ± 0.01	1.5 ± 0	1.02 ± 0.02	68.12 ± 0.04
	5	0.32 ± 0.01	1.5 ± 0.01	0.81 ± 0.01	53.88 ± 0.03
	7.5	0.32 ± 0.02	1.5 ± 0	0.82 ± 0.01	54.62 ± 0.03
	10	0.31 ± 0.01	1.5 ± 0.01	0.78 ± 0.01	52.05 ± 0.02
P6CCG	2.5	0.29 ± 0.01	1.5 ± 0.01	0.72 ± 0.02	48.32 ± 0.04
	5	0.29 ± 0.02	1.5 ± 0	0.71 ± 0.02	47.54 ± 0.05
	7.5	0.30 ± 0.02	1.5 ± 0.01	0.74 ± 0.04	49.40 ± 0.07
	10	0.30 ± 0.01	1.5 ± 0.01	0.72 ± 0.01	48.30 ± 0.02

**Table 5:** Irritation scores of samples evaluated by hen’s egg test-chorioallantoic membrane assay (HET-CAM).

Samples	Test Concentration		
	2 wt%	4 wt%	100 wt% (pure)
PGCE-1	5.1	8.4	-
P6CCG	9.9	14.1	-
Olive oil PEG-8 esters	6.1	8.2	-
Poloxamer 184	4.0	5.2	-
0.1 mol/L NaOH	-	-	18.5
0.9% NaCl	-	-	0

## Supplementary Materials

The following are available online at https://www.mdpi.com/2073-4360/12/2/294/s1, Figure S1: Photos of chicken embryo chorioallantoic membrane in contact with (a) 4 wt% PGCE-1 solution, (b) 4 wt% P6CCG solution, (c) 4 wt% olive oil PEG-8 esters, (d) 4 wt% Poloxamer 184, (e) 0.9 wt% NaCl solution, and (f) 0.1 mol/L NaOH solution for 60 s.
