# Supplementary material for: Synthesis, Characterization, Self-Assembly, and Irritation Studies of Polyglyceryl-10 Caprylates
Source: Polymers (Basel). 2020 Feb 2;12(2):294. doi: 10.3390/polym12020294 (PMC7077386; doi:10.3390/polym12020294)
Supplement: Supplementary file 1 [file polymers-12-00294-s001.pdf]

Synthesis, Characterization, Self-assembly, and Irritation Studies of Polyglyceryl-10 Caprylates

Guangyan Zhang, Chenhui Bao, Kaiqiao Fu, Yaolin Lin, Tianlong Li and Huping Yang

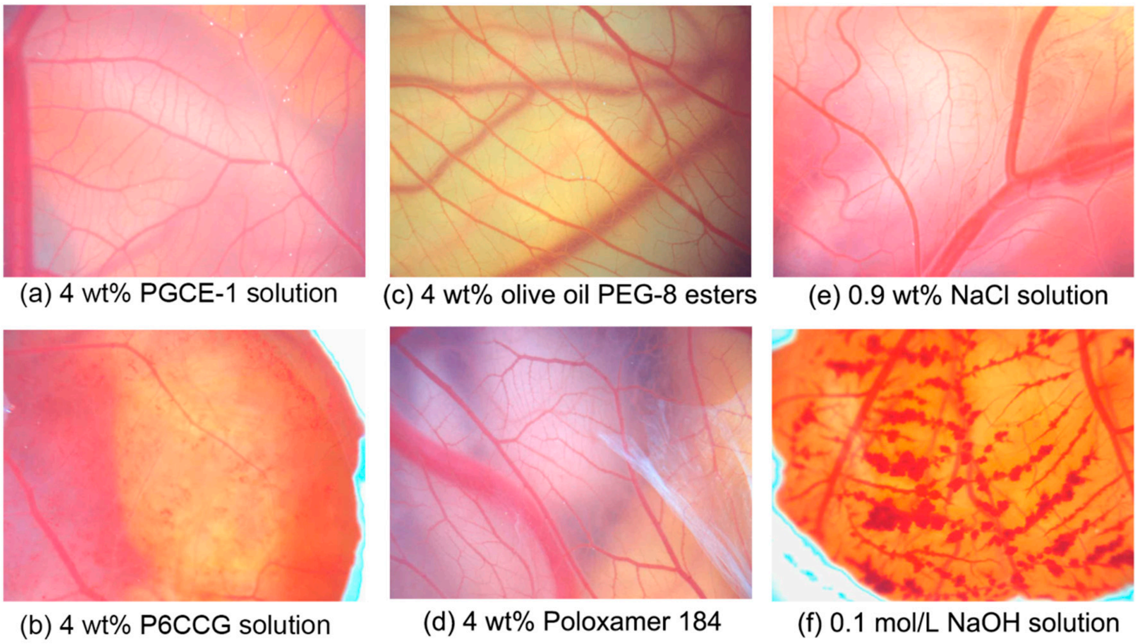

Figure S1. Photos of chicken embryo chorioallantoic membrane in contact with (**a**) 4 wt% PGCE-1 solution, (**b**) 4 wt% P6CCG solution, (**c**) 4 wt% olive oil PEG-8 esters, (**d**) 4 wt% Poloxamer 184, (**e**) 0.9 wt% NaCl solution, and (**f**) 0.1 mol/L NaOH solution for 60 s.
